# Understanding the formulation of non-communicable disease policies in Nepal: a qualitative study

**DOI:** 10.1093/heapol/czag048

**Published:** 2026-04-08

**Authors:** Anju Vaidya, Padam Simkhada, Edwin van Teijlingen, Andrew Chee Keng Lee

**Affiliations:** School of Human and Health Sciences, University of Huddersfield, Queensgate, HD1 3DH, United Kingdom; Research and Innovation Office, University of Chester, Parkgate road, Chester, CH1 4BJ, United Kingdom; Faculty of Health, Environment and Medical Sciences, Bournemouth University, Bournemouth Gateway Building (5th floor), St Paules Lane, Bournemouth, BH8 8GP, United Kingdom; Public Health Office, Sheffield Centre for Health and Related Research, University of Sheffield, Level 2 Regent Court, Regent Street, Sheffield, S1 4DA, United Kingdom

**Keywords:** advocacy, evidence-based policy, policy process, non-communicable diseases, political prioritization

## Abstract

Few policies have focused specifically on the growing burden of non-communicable diseases (NCDs) in low- and middle-income countries. Health policy formulation plays a vital role in the allocation of resources to implement effective interventions and reforms; hence, a nuanced understanding of the health policy formulation process is essential. However, there is limited evidence about the process through which NCD policies were formulated in Nepal. This study used Kingdon’s multiple streams framework to explore how NCDs were recognized and prioritized, how policy alternatives were decided, how policy windows were opened, and which contextual factors influenced the policy formulation process. A qualitative case study approach was applied to gain a comprehensive understanding of the formulation of major NCD-related policies in Nepal. Semi-structured interviews were conducted with 12 key stakeholders and policy documents were analyzed using framework analysis. The NCDs were gradually recognized and prioritized through the convergence of global and local evidence, sustained advocacy, and international commitments. Policymakers encountered several challenges, such as competing health priorities, the chronic nature of NCDs, donor preferences for communicable diseases, financial constraints, and multisectoral complexities of NCDs. The Package of Essential Non-communicable diseases interventions were adopted as a policy alternative, informed by global evidence, World Health Organization recommendations, and lessons from other countries. While coordinated efforts by stakeholders brought the problem, policy, and politics streams together, the role of policy entrepreneurs was found to be less relevant in Nepal’s context. The findings highlight the need to consider external influences while conducting similar studies in low- and middle-income countries. Further research is needed on strategies to address persistent structural and financial challenges in NCD policy formulation.

Key messages

- Global and local evidence on non-communicable diseases (NCDs), persistent advocacy, and international commitments opened the window of opportunity, resulting in political prioritization and formulation of NCD policies in Nepal.
- Kingdon’s multiple streams framework offers insights into the complex and multifaceted aspects of the NCD policy formulation process.
- External factors such as persistent advocacy and support from the World Health Organization and international commitments to NCDs significantly contributed to the successful formulation of relevant policy in Nepal. This suggests the importance of assessing external influences in low- and middle-income countries policy processes.
- Empirical evidence generated in Nepal offers transferable lessons for understanding NCD policy formulation in other low- and middle-income countries.

## Introduction

The epidemic of non-communicable diseases (NCDs) is a global public health issue. Many countries have experienced a shift from communicable diseases to NCDs, while others face a dual disease burden (Roth et al. 2018). The latest global estimate for 2021 is that 43 million deaths occurred due to NCDs, some 75% of all global deaths. Most NCD-related global deaths were due to cardiovascular disease (CVD), cancer, chronic obstructive pulmonary disease, and diabetes mellitus (Li et al. 2025, World Health Organization 2025). NCDs have a disproportionate impact globally, with 80% of premature deaths occurring in low- and middle-income countries (LMICs) (World Health Organization 2025). In addition to premature deaths, the rising NCD prevalence contributes to increased poverty and poses a threat to national economies (Department of Health Services 2023).

Like many other LMICs, the burgeoning trend of NCDs holds true in Nepal where 71.1% of all deaths were due to NCDs in 2019, rising from 51% in 2010 (Nepal Health Research Council et al. 2021, Department of Health Services 2023). The deaths in Nepal were mainly due to four major NCDs: CVD, chronic obstructive pulmonary disease, cancers, and diabetes mellitus, accounting for 24%, 16%, 11%, and 4% respectively (Nepal Health Research Council et al. 2021). The growing NCD burden is closely linked to increasingly prevalent behavioural risk factors, including smoking, alcohol use, unhealthy diets, and physical inactivity, accounting for 17%, 7%, 97%, and 8% respectively (Dhimal et al. 2019, Gyawali et al. 2020). The rise in NCDs is associated with a decreased quality of life, catastrophic health expenses, and loss of productivity (Government of Nepal 2014), and places additional pressure on an already challenged health system (Sharma et al. 2019).

In response, the Government of Nepal (GoN) has introduced several policy initiatives to address NCDs. Nepal became a signatory to the 2011 United Nations (UN) Political Declaration on the Prevention and Control of NCDs. Subsequently, the National Health Policy was revised in 2014 which explicitly recognized NCDs as a significant public health concern (Ministry of Health and Population 2014). This led to the development of the Multisectoral Action Plan for Prevention and Control of NCDs (2014–2020) as a major policy initiative introduced by the GoN to guide the implementation of NCD-related activities via a multisectoral approach (Government of Nepal 2014). The latter received technical support from the World Health organization (WHO) and financial support from the WHO and Russia (Government of Nepal 2014). The Package of Essential Non-communicable diseases (PEN) interventions were adopted in 2015, followed by endorsement of the PEN protocol in 2016. The PEN programme features four simplified clinical protocols (Table 1) designed specifically for primary health care settings, enabling healthcare workers to effectively identify individuals at risk, manage diagnosed individuals, and promote behavioural change (World Health Organization 2013b, World Health Organization et al. 2019b, Department of Health Services 2023, 2025). The PEN interventions were implemented in 77 districts in Nepal (Department of Health Services 2024, 2025). Subsequent revision of the National Health Policy in 2019 further emphasized multisectoral coordination and integrated health system approaches (Ministry of Health and Population 2019). In 2022, the action plan was revised as the Multisectoral Action Plan for prevention and control of NCDs (2021–2025). This policy was yet to be endorsed during the data collection period, and therefore, its policy formulation process is not explored here. An overview of the NCD policies is presented in Table 1.

**Table 1:** Overview of NCDS policies.

Policies	Focus
National Health Policy 2014	Focuses on universal access to health services, basic health services, health financing, governance, and human resources.
Multisectoral action plan for prevention and control of NCDs (2014–2020)	Mainly focuses on prevention of NCDs, including lifestyle and behaviour change (tobacco and alcohol control), strengthening primary health care, monitoring and supervision, and multi-sectoral coordination.
National Health Policy 2019	Focuses on more comprehensive health services such as Universal Health Coverage, integrated services (promotive, curative, rehabilitative, and palliative), financing, governance, and health information systems.
Package of essential non communicable disease (PEN) intervention at primary health service setting: PEN training trainee’s manual	PEN interventions have four simplified protocols. Protocol 1: heart attack, stroke and kidney disease prevention via integrated management of diabetes and hypertension. Protocol 2: health education and counselling on healthy behaviours. Protocol 3: chronic obstructive pulmonary disease and asthma management. Protocol 4: assessment and referral of women with suspected cancer (breast and cervix). The main objectives of PEN interventions are: (i) strengthen the health system to prevent and control NCDs and their risk factors through PHC services; (ii) strengthen capacity and coordination at the national and local levels for effective NCD prevention and control; and (iii) reduce NCD-related modifiable risk factors and social determinants through health promotion initiatives.

Despite several policy developments, little is known about how NCD policies were formulated. Understanding the policy formulation process, such as who the key actors were, how problems were prioritized, and what factors facilitated or constrained the processes, is essential for strengthening future policy responses (Unwin et al. 2017). A review of the literature found only one paper from Nepal exploring the policy formulation process, particularly for CVDs (Pradhan et al. 2023). Given Nepal’s unique political and administrative context, decentralized government structure, constrained health financing, competing health priorities, and reliance on external funding, it is unclear how policy-making mechanisms operate in practice.

This study addresses this gap by examining the NCD policies formulation process in Nepal and exploring how NCDs were recognized and prioritized, how policy alternatives were selected, how political factors shaped decision-making, and which factors facilitated or hindered the policy formulation process. Using a qualitative case study design, the study provides insights into the dynamics of NCD policies formulation in Nepal and contributes to broader understanding of policy formulation processes in LMIC contexts.

### Theoretical framework

This article draws on Kingdon’s multiple streams framework (MSF) to examine the policy formulation processes. It focuses on how the three streams (problem, policy, and politics) are brought together, often by policy entrepreneurs, during the window of opportunity to formulate the policy. The problem stream describes how health problems are recognized and prioritized by the policy actors through indicators, focusing events, and feedback from existing programmes and routinely monitored activities (Kingdon 1984). The policy stream describes the process through which potential solutions and strategies to solve pressing problems are discussed and finalized among policymakers (Kingdon 1984). The politics stream illustrates how the political environment, including political actors and their preferences, institutional context, and national and international influences shape the decision-making process (Jones et al. 2015). The framework describes how the coupling of these three independent streams takes place and opens the window of opportunity for issues to get into the policy agenda and how policy is formulated (Kingdon 2011). Although the framework was originally developed for the USA, it has been increasingly used in LMIC settings to understand health policy processes. Strengths and limitations of the framework are described in supplementary file S1, see online supplementary material.

### Methodology

A qualitative case-study design explored the process of NCD policy formulation in Nepal, including enabling and constraining factors contributing to the policy process. The case-study design helped to clarify the complex phenomenon of the NCD policy formulation process in its natural context by using multiple approaches (Yin 2014). Triangulation of data using multiple sources enhanced the study’s trustworthiness.

Data were collected using three approaches: literature review, document review, and semi-structured key informant interviews. The literature review was conducted systematically from two databases (PubMed and CINAHL) for publications between 2011 and 2022, using inclusion and exclusion criteria (supplementary file S2, see online supplementary material). The search was supplemented by hand-searching reference lists of the retrieved articles, as well as searches of Google Scholar and the WHO Institutional Repository for Information Sharing. The document review was used as a complementary source of evidence to gather further relevant information. Official documents related to NCD policies and policy process activities, including government reports, policy plans and strategies, and regulatory documents (Table 2) were reviewed to contextualize the policy process.

**Table 2:** List of reviewed documents.

Author	Name of the document
World Health Organization 2013a	Global action plan for the prevention and control of noncommunicable diseases 2013–2020.
World Health Organization 2013b	Implementation tools: Package of essential noncommunicable (PEN) disease interventions for primary health care in low-resource settings.
Ministry of Health and Population 2014	National Health Policy 2014
Government of Nepal 2014	Multisectoral Action Plan for Prevention and Control of Non-communicable Diseases (2014–2020)
Government of Nepal 2015	Constitution of Nepal
Ministry of Health and Population 2019	National Health Policy 2019
World Health Organization et al. 2019a	Package of Essential Non communicable Disease (PEN) intervention at primary health service setting: PEN training trainee’s manual
Department of Health Services 2023	Annual Report (2021/22)
Department of Health Services 2024	Annual Health Report 2079/80
Department of Health Services 2025	Annual Health Report 2080/81

Semi-structured key informant interviews were then conducted. The interview guide was developed based on literature review, research objectives, discussions with research team members, and informal discussions with experts, and framed using key concepts of Kingdon’s MSF.

NCD policy documents did not name the policy actors involved in the policy formulation, therefore, policy actors were purposively selected for interview. The information officer of the Ministry of Health and Population (MoHP) was contacted to gather information about the stakeholders involved in the policy formulation process. Furthermore, individuals working in the NCD area were searched on Google and contacted. They were asked about their involvement in the process, and those who participated in the process were purposively selected. Snowball sampling was also employed (Noy 2008) to recruit other relevant participants from the network of policy actors. This network of policy actors was the focus of the study, which was one of the reasons for selecting these sampling techniques (Clark et al. 2021). Of 17 individuals contacted, 12 were recruited to the study, 1 declined to participate, and 4 were not involved in the policy process.

Semi-structured interviews were conducted in 2021, lasting 30–60 min. As the policy formulation process involves different stages (e.g. problem and policy option identification, consultation, advocacy, and decision-making), the involvement of participants involved in these stages was taken into consideration when selecting participants.

Key informants were provided with information sheets and consent was obtained prior to the interviews. An iterative approach was followed, and reflections from each interview were incorporated into subsequent interviews, e.g. some participants mentioned the important role of WHO advocacy and adoption of the PEN programme as a policy option. This was further explored with subsequent participants to obtain further understanding of the advocacy process. This approach not only facilitated a deeper understanding of the process but also enabled validation of the findings. Data were collected until theoretical saturation was reached, i.e. no new information emerged from further interviews (Padgett 2017). We also adopted the concept of ‘information power’ to determine the sample size of this study (Malterud et al. 2016) (supplementary file S3, see online supplementary material). We acknowledge that there is a growing question regarding genuineness, bias, and completeness about collected data, along with the difference found between what people say and what they do (Hammersley 2008, Padgett 2017). To enhance trustworthiness, interview data were integrated, compared, and contrasted with data from other sources (Yin 2014). Interviews were audio-recorded, except for one, where handwritten notes were taken, and transcribed verbatim.

Framework analysis was applied which included five key steps: (i) data familiarization; (ii) framework identification; (iii) indexing of data; (iv) organization of indexed data into a format; and (v) mapping and interpreting data to find key patterns and abstractions (supplementary file S4, see online supplementary material). NVivo 14 (QSR International) was used for data management and analysis.

## Results

The findings are presented based on key components of Kingdon’s MSF (problem stream, policy stream, politics stream, policy entrepreneurs, policy windows). The study also presents information on the involvement of various actors in the policy formulation process (Table 3).

**Table 3:** Participant characteristics.

Type of respondent	Total number
Government actors	8
Ministry of Health and Population (1)	
Government organization (2)	
Research/academic institutions (3)	
Hospital (2)	
Non-government actors	4
International organization (1)	
NGOs/civil society organization (2)	
Research/academic institutions (1)	

### Actors

Although the actors are not identified as one of the key components of Kingdon’s MSF, each of the three streams is driven by their actions and interactions. Providing information about which actors were involved (or excluded) and how they were selected helps to reveal underlying power dynamics, institutional constraints, and network influences, shaping the policy process (Oltmer and Löhr 2025). Including this component, therefore, allows for a more comprehensive and nuanced application of the framework.

#### Stakeholder engagement

A diverse range of stakeholders participated in the NCD policy formulation, including representatives from the MoHP, other ministries, non-government organizations (NGOs), international organizations, professional societies, academics, researchers, and clinicians, including currently working and retired officials (Table 3).

Most participants highlighted that the MoHP played a central coordinating role, guiding consultations and consolidating input from various sectors. However, a few participants perceived the stakeholder selection and engagement process as informal and influenced by personal and political relationships rather than systematic expertise-based selection. One participant reflected:

‘If the Health Minister or the Health Secretary knows me, he will call me for consultation regarding NCD issues and policies.’ (Civil society representative)

This resulted in limited engagement of key stakeholders such as NGOs, researchers working in the NCD area, people living with NCDs, and frontline implementers. The exclusion of these actors was perceived to have constrained the inclusiveness and diversity of perspectives in the policy process.

Participants (researchers and clinicians) further mentioned that policymakers viewed clinical professionals as primary subject experts and prioritized their involvement in the policy process, while those engaged in academic or NCD-related empirical research were often overlooked for consultation.

Despite recognition of the multisectoral nature of NCDs, participants (MoHP representatives and international organization representative) reported challenges in multisectoral coordination during the policy formulation process. Engagement of non-health ministries in the policy formulation process was constrained by competing priorities, limited awareness about the multisectoral nature of NCDs, and perceptions that NCDs were solely a health responsibility and did not fall within the scope of other ministries. These findings highlight persistent institutional and perceptional barriers to shared accountability and effective cross-sectoral collaboration in the policy formulation process.

### Problem stream: recognition and prioritization of NCDs

Some participants (International Non Government Organization representative, researcher, health activist) noted that NCD recognition emerged gradually, initially through tobacco control efforts, particularly the formulation of tobacco control policies in 2010 (Tobacco Control Laws 2014). However, only tobacco control issues gained political prioritization initially while other NCDs received little attention. A few clinicians reported that although they recognized increasing NCD-related deaths through their experience, the issue could not gain political attention initially due to a lack of local evidence.

#### Role of indicators

Over time, participants (clinician, civil society representative, and researchers) highlighted that global and local evidence, particularly the Global Burden of Disease study and national STEPS (WHO STEPwise approach to NCD risk factor surveillance) survey, were crucial in demonstrating the increasing NCD burden and attracting political attention. Evidence generated by government institutions was perceived as a credible indicator and influenced policymakers’ perceptions because of their close relationship with the MoHP (Aryal et al. 2015).

Barriers to research uptake by policymakers (clinician and researcher), included limited efforts by researchers to share evidence with policymakers in an understandable manner and the irrelevant timing of evidence dissemination.

‘We, as researchers, were not able to present policy research in an understandable manner to the policymakers. There was a lack of ability to push this issue forward among researchers… The focus of the researcher is on how he can conduct the research, collect data, and perform analysis. Similarly, we need to know what the time points are for the uptake of that evidence. For example, suppose I am a policymaker. You come and tell me about your study findings. I will say that it is good. But how am I going to use these findings? So timing is also particularly important.’ (Researcher)

#### Advocacy and issue framing

Participants (researchers, MoHP representative, WHO representative, public health professional) mentioned that persistent advocacy by national and international stakeholders, including the WHO and the NCD Alliance, further contributed to problem recognition. However, advocacy activities were reported to be often individualized, sporadic, lacked a structured plan, and patient voices were largely absent.

Participants (researcher and civil society representative) mentioned that NCDs were initially framed as a health issue requiring hospital-based approaches. Over time, with increasing realization of the need to prevent NCDs, the framing shifted to lifestyle, emphasizing public awareness, education, and promotion of adopting healthy behaviours (Government of Nepal 2014, Ministry of Health and Population 2019).

‘People’s responsibility to keep themselves healthy and healthy lifestyle shall be promoted through health awareness programmes.’ (Ministry of Health and Population 2019, p.27)

#### Challenges to NCD prioritization

Participants (researchers) noted several factors impeding NCD prioritization. The government’s attention to existing health issues, such as communicable diseases, maternal and child health, and commitment to Millennium Development Goals (MDGs) constituted a key challenge to getting NCDs on the policy agenda.

‘At that time, the target of the government and non-government organization was on the MDGs. There were no NCDs in the MDGs. So, convincing policymakers about the situation of NCDs was incredibly challenging.’ (Researcher)

The chronic nature of NCDs requiring long-term interventions was cited by some (researcher and public health professional) as another reason for NCD’s low priority.

‘When people become ill immediately and die in a short interval, it grabs the attention of the government, politicians, and policymakers. In case of NCDs, as the person becomes old, the prevalence of NCDs increases, and it requires management for long durations.’ (Public health professional)

Participants (clinician, researcher, public health professional) highlighted the influence of funding on NCD prioritization, with donor support skewed towards communicable diseases. Because of Nepal’s high dependency on external funding, donor priorities often shaped the policy agenda, leaving NCDs with a lower priority during the policy formulation process.

Despite these challenges, participants (clinician and INGO representative) reported that collective and sustained advocacy by a wide range of stakeholders, including the MoHP, senior bureaucrats, WHO, clinicians, and researchers working in the NCD area contributed to NCD prioritization.

### Policy stream: selection of policy alternatives

PEN interventions were adopted as a strategy to prevent and control NCDs (Government of Nepal 2014). Most participants highlighted the significant influence of the WHO in shaping policy decisions to adopt PEN. The findings demonstrate the WHO’s role in advocating for the PEN programme through synthesizing and sharing evidence of successful implementations in other countries. Through continuous engagement, the WHO highlighted the cost-effectiveness and feasibility of the PEN programme within Nepal’s resource-constrained context and facilitated informed decision-making. The institutional relationship and trust between the WHO and MoHP also influenced this decision.

Half of the participants noted that the formulation process for policy documents, e.g. Multisectoral Action Plan for prevention and control of non-communicable diseases (MSAP) (2014–2020), was guided by global guidelines such as the Global NCD Action Plan, which was later contextualized to Nepal (World Health Organization 2013a, Government of Nepal 2014).

#### Financial challenges

Developing a sustainable financial strategy emerged as a key challenge for policymakers. Despite realizing the necessity of addressing NCDs, participants (MoHP representative, clinician, researcher) noted that balancing technical feasibility with financial sustainability was a persistent concern.

‘We realized that it was necessary. We were trying to strike a balance between technical and financial feasibility. So, that was the concern to begin with because once you start the range of free medicines, the public funding, public expenditure will go up, and that responsibility will have to be taken by the central government.’ (Clinician)

#### Health system capacity and implementation challenges

Another concern raised during policy selection included integration into routine primary healthcare services. Participants (researcher, INGO representative) noted that while primary healthcare staff could manage communicable diseases, they lacked the capacity and training to manage NCDs effectively. Integrating PEN within the primary health system required task shifting, training, and systemic restructuring (Government of Nepal 2014), which were perceived as major challenges.

### Politics stream: national and international commitments

International engagement played a crucial role in influencing political support. Participants (MoHP, INGO representatives, public health professional) highlighted that Nepal’s participation in global health events, particularly in the 2011 Political Declaration of High-Level Meeting on the Prevention and Control of NCDs, played a significant role in catalyzing policy response in favour of NCD prevention and control (United Nations 2011, Government of Nepal 2014).

Participants (MoHP, INGO representatives, public health professionals) noted the significant influence of commitment to the Sustainable Development Goals (SDG) on NCD recognition in the national agenda that catalyzed policy response (Ministry of Health and Population 2019). The NCD policies also aligned with Nepal’s prevailing laws and constitutional provisions on health (Government of Nepal 2015, Ministry of Health and Population 2019).

‘This policy is also imperative to address the national and international commitments made by Nepal and to achieve the Sustainable Development Goals…’ (Ministry of Health and Population 2019, p.22)

Most participants recognized that advocacy by both national and international actors helped sensitize political leaders to the growing NCD burden and the importance of addressing NCDs. It strengthened political support despite donor dependence continuing to influence priorities.

### Policy entrepreneurs and opening of the policy window

Most participants did not explicitly identify policy entrepreneurs, although the WHO and selected national actors played significant entrepreneurial roles by raising NCD awareness, promoting feasible solutions, and coupling the three streams.

The findings demonstrated that the convergence of the three streams, problem, policy, and politics, created a conducive environment to open the policy window for NCD policy formulation in Nepal. In the problem stream, NCD was recognized as a major public health concern, supported by evidence from national and global studies. The availability of feasible and evidence-informed policy alternatives, such as the PEN interventions, enabled its adoption in the policy stream. The politics stream gained momentum through participation in international health fora, international commitments like SDGs, and advocacy from national and international stakeholders. These three streams intersected and opened a policy window that enabled policymakers to prioritize NCDs and the PEN programme.

## Discussion

**Figure 1:**
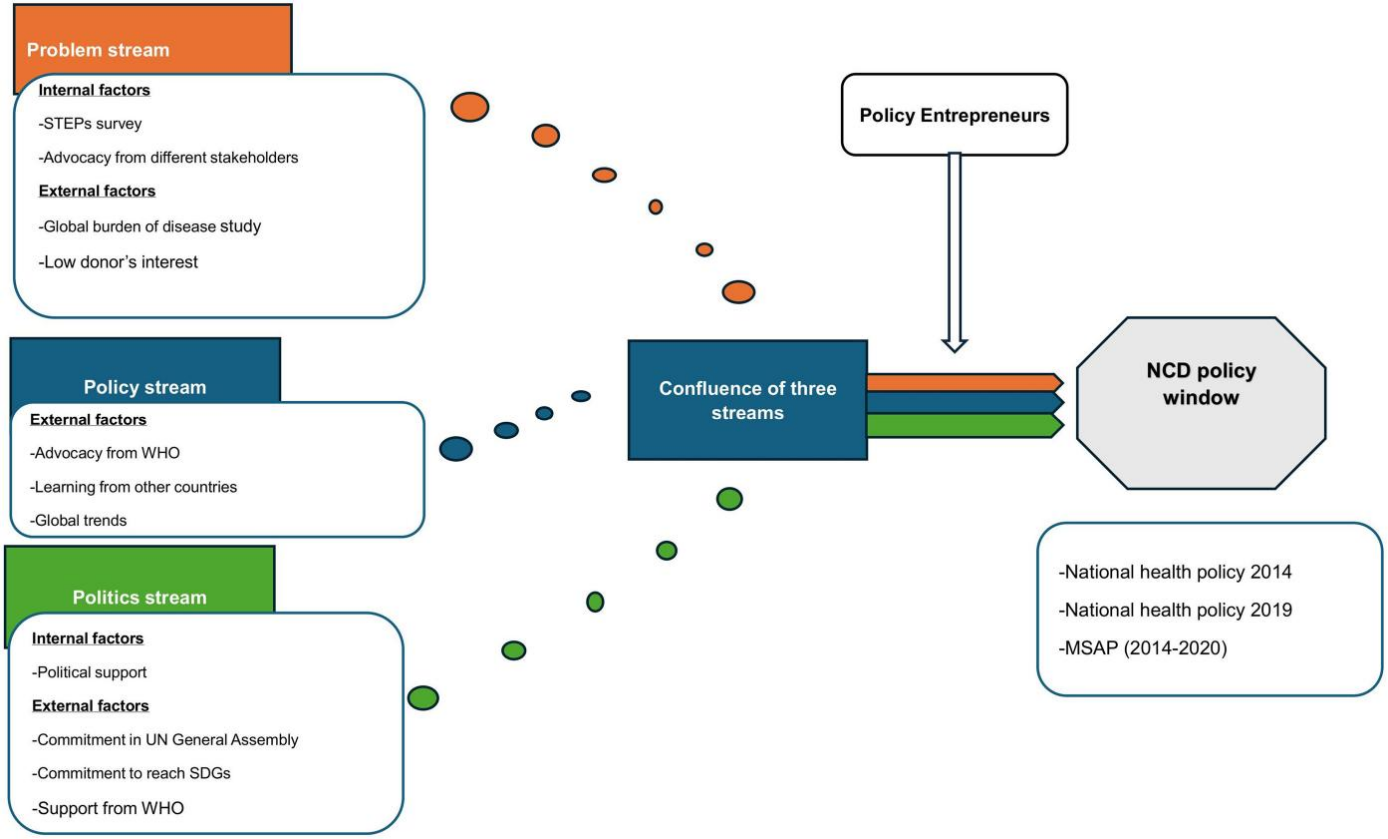
NCD policy formulation process in Nepal (Source: Kindon 2011).

This study examined how NCDs became a national priority, how they gained political prioritization, how policy option(s) were decided, how political factors, including other factors, facilitated or hindered the process, and how the three streams converged to open a policy window and formulate the policy. Applying Kingdon’s MSF generated insights into the dynamics of the policy formulation process in Nepal and highlighted areas where the framework may require refinement for LMICs (Fig. 1) (Kingdon 2011). Study limitations are presented in supplementary file S5, see online supplementary material.

Overall, the findings demonstrated that the convergence of global commitments, global guidance particularly from the WHO, combined with credible national evidence and increasing political attention, strengthened policymakers’ perception of the urgency and feasibility of policy actions. This alignment opened a policy window, resulting in political prioritization of NCDs and adoption of the PEN programme.

However, several challenges were faced during the process. Competing health priorities, the chronic nature of NCDs requiring long-term interventions, limited donor investment in NCDs, financial constraints, and weak multisectoral coordination hindered the process. Despite these challenges, credible indicators, advocacy from national and international actors, and international commitments facilitated political prioritization of NCDs, leading to NCD policy formulation.

### Stakeholder involvement and multisectoral coordination

Although a wide range of stakeholders were consulted during the policy formulation process, their engagement was neither systematic nor expertise driven. Meaningful stakeholder participation was constrained by political relationships, limited use of research expertise, and weak multisectoral collaboration mechanisms. The participants highlighted insufficient engagement of key stakeholders, such as researchers, civil society, individuals living with NCDs, and frontline implementers, compromising the policy formulation process.

Clinicians were preferred over researchers for consultation during the policy formulation process, limiting balanced participation. Policymakers’ emphasis on clinical perspectives over research evidence reflects a narrow understanding of expertise, potentially limiting evidence-informed decision-making (Lange et al. 2022, Stephens et al. 2024). The exclusion of the engagement of individuals living with NCDs further limited incorporation of experiential knowledge in the policy formulation process, which could contribute meaningfully to the design of interventions (Shiroya et al. 2019) and strengthen citizen-focused policy design (Health and Global Policy Institute & NCD Alliance 2022, Singh et al. 2023, You et al. 2024). In Nepal’s context, this exclusion may stem from policymakers’ assumption that public involvement would be of little value to the policy process (Stephens et al. 2024).

Although the PEN programme was intended to be implemented at primary healthcare facilities, there was limited involvement of frontline implementers, also referred to as street-level bureaucrats (frontline staff who deliver services to the public) (Buse et al. 2012), in the policy formulation process. Exclusion of these key stakeholders may weaken policy design and implementation fidelity (Liu et al. 2010, Sanni et al. 2019). These dynamics underscore the need for a more structured and evidence-informed stakeholder engagement plan that can help ensure engagement of an adequate and broad array of relevant stakeholders in future NCD policy formulation, further ensuring policy acceptability and effectiveness among stakeholders (Lane et al. 2020).

The findings highlighted persistent institutional and perceptual barriers to effective multisectoral coordination. Despite the MoHP’s efforts to engage non-health ministries during the policy formulation process, coordination, shared accountability, and sustained participation remained a significant challenge. These challenges were attributed to competing priorities and perceptions that NCDs were solely a health sector responsibility, undermining a whole-of-government approach. Similar coordination challenges were reported in African LMICs, often due to a lack of clear coordination mechanisms to guide different inter-sector collaboration (Juma et al. 2018a).

These findings highlight the need for structured stakeholder engagement strategies. Engaging diverse sector stakeholders such as civil society and non-health ministries in the policy formulation process from the start may enable a trusting relationship with the legislators and allow a ‘whole of society, whole of government approach’ (Loffreda et al. 2024). Similarly, capacitating leadership across sectors and all government levels to cultivate champions in different sectors can lead to better policy coherence and accountability among the multiple sector stakeholders, which is essential to effectively respond to NCDs (Juma et al. 2018a; Loffreda et al. 2024). This approach can enhance their knowledge about the issue, enabling all to engage more effectively in the policy process (Latu et al. 2018). Strengthening these mechanisms is essential for effective multisectoral coordination and sustainable NCD governance in Nepal.

### Problem stream: the role of evidence

Recognition of NCDs as a public health problem was a gradual process. Initially, limited local evidence constrained political prioritization. However, the progressive increase in NCD-related global and local evidence, particularly the Global Burden of Disease study and STEPS survey, sensitized policymakers to the rising NCD burden and drove political attention. These findings align with other LMIC studies (Faraji et al. 2015, Mukanu et al. 2017, Unwin et al. 2017, Ndinda et al. 2018, Wickramasinghe et al. 2018, Amerzadeh et al. 2020, Nepal Health Research Council et al. 2021, Lange et al. 2022, Pradhan et al. 2023) and support the relevance of the problem stream in Nepal (Kingdon 1984).

The STEPS survey findings were perceived as highly credible as the study was conducted by a government organization closely affiliated with the MoHP and supported by the WHO. These findings echo studies from Iran and a recent systematic review (Amerzadeh et al. 2020, Loffreda et al. 2024). Evidence produced by institutions with trusted relationships with the MoHP appeared more influential in the policy formulation process than research from independent researchers. While institutional trust and proximity enhanced evidence uptake, reliance on government-affiliated evidence may narrow the scope of evidence. A South African study highlights the importance of consistent and comparable data in understanding NCD patterns, informing policy decisions, and achieving reduction targets (Ndinda et al. 2018). Strengthening the utilization of diverse, high-quality local research could enhance the inclusivity and robustness of future NCD policymaking. Moreover, involving MoHP officials or policymakers in research design could enhance their awareness and appreciation of evidence, thereby improving research uptake.

Low research uptake of NCD research by policymakers was also attributed to ineffective communication by researchers and evidence sharing at inopportune times (Barreto et al. 2024). Addressing these barriers requires targeted strategies to tailor evidence dissemination to policymakers’ needs, such that accessibility and timely availability is ensured.

### Policy stream: adopting the PEN programme

The decision to adopt the PEN programme was largely influenced by global evidence and WHO advocacy. The WHO advocated PEN adoption and its guidance ensured that Nepal’s policy was evidence informed. These findings were consistent with studies from Afghanistan (Lange et al. 2022). The institutional trust between the WHO and MoHP further enhanced policymakers’ perceived legitimacy of PEN as a credible and feasible policy option. These findings aligned with Kingdon’s policy stream, where policy communities develop viable solutions to the identified problems (Kingdon 1984).

Unlike contexts like Romania, where policy adoption decision was guided by local evidence, in Nepal it was mainly based on global evidence and WHO recommendations. While external support accelerated policy adoption, it also reflects capacity limitations among national actors to formulate the policy and reliance on external stakeholders. Similar patterns were observed in other LMICs, underscoring the necessity to strengthen domestic research capacity, national ownership, contextual adaptation of strategies, and an evidence-informed policy culture for long-term sustainability and policy success (Mamka Anyona and de Courten 2014, Essue and Kapiriri 2018, Wang et al. 2021).

A balanced and effective collaboration between global and national actors, where international support complements local evidence, is essential to ensure that policy decisions are feasible, contextually relevant, and align with global best practices. This aligns with Lange et al. (2022), who highlighted the importance of aligning global strategies like PEN with national needs for effective and sustainable policy implementation.

Financial feasibility emerged as a key concern among policymakers while deciding the policy option in Nepal due to persistent donor dependency and limited domestic funding. Challenges in balancing technical feasibility and financial sustainability posed risks for long-term sustainability of policy interventions, echoing challenges across LMICs (Essue and Kapiriri 2018, Juma et al. 2018b). Strengthening financial planning, diversifying funding resources, and embedding NCD interventions within national budgets could enhance sustainability and reduce reliance on external sources.

### Politics stream: international commitments and political will

Political prioritization of NCDs intensified following Nepal’s participation in the 2011 UN Political Declaration of High-Level Meeting on Prevention and Control of NCDs. This event initiated global momentum on NCDs by bringing NCDs to the forefront in global discussions and influencing member states, including the GoN, to commit to national-level policy actions (United Nations 2011). The event therefore marked a milestone that facilitated recognition of NCDs as a national priority and formulation of the NCD policies in Nepal. Furthermore, international commitments, including the SDGs, reinforced NCD prioritization and policy initiatives (United Nations 2015), as also observed in Iran, Zambia, and Barbados (Mukanu et al. 2017, Unwin et al. 2017, Amerzadeh et al. 2020). This highlights the vital role of global health governance and international commitments in shaping national priorities and NCD policy formulation in LMICs.

Although the government’s participation in global events demonstrated strong political commitment, Nepal’s dependence on donors for financial and technical assistance constituted a challenge. Donor priorities often focusing on communicable diseases influenced national priorities. Limited donor funding for NCDs impeded NCD policy expansion and implementation, consistent with findings from Africa (Juma et al. 2018b, Oladepo et al. 2018). Similar trends were witnessed elsewhere, where policy priorities aligned with donor-funded areas, demonstrating how donor dependency shapes national policy directions (Essue and Kapiriri 2018, Wang et al. 2021). Strengthening domestic resource mobilization, continued advocacy, and fostering national ownership, are therefore essential to translating political commitments into effective and sustained policy outcomes.

### Policy entrepreneurs

The findings showed that the concept of ‘policy entrepreneurs’ among policymakers in Nepal was relatively unclear and not widely recognized. Although stakeholders did not formally identify themselves as policy entrepreneurs, their actions demonstrated entrepreneurial behaviour. For example, the WHO actors, alongside national ones, played vital roles in raising awareness of NCDs and the need to address them (according to Kingdon—‘soften up’) and recommended policy options that were technically and financially feasible for the local context and acceptable to policymakers (‘coupling’) (Kingdon 1984). Similar dynamics were witnessed in Belarus and Barbados (Unwin et al. 2017, Famenka et al. 2018). Recognizing such actors’ roles may enhance future policy processes.

### Application of MSF: emergence of a ‘global stream’

The application of Kingdon’s MSF offered insight into the multifaceted aspects of the NCD policy formulation process in Nepal. A key finding was the significant influence of external factors across all three streams: problem, policy, and politics (Fig. 1). Global evidence, participation in international health events, global commitments, and WHO advocacy shaped problem recognition, policy selection, and political momentum. The emergence of what may be conceptualized as a ‘global stream’, in addition to Kingdon’s three streams, was evident in Nepal. Nepal’s participation in international fora, the SDGs, the WHO’s advocacy, technical support, and recommendations, particularly regarding PEN adoption, were key drivers that opened the policy window. These findings demonstrated the crystallization of a distinct ‘global stream’ that interacted with the domestic policy processes during the NCD policy formulation. Understanding the role of external factors is, therefore, essential when assessing the policy formulation process in LMICs like Nepal, where international actors often play a significant role in shaping national priorities.

Kingdon’s MSF was primarily developed to understand the policymaking process in the USA, focusing mainly on the role of domestic actors, and does not explicitly account for influence of external dynamics. In Nepal, like many LMICs, global governance structures significantly influenced national policymaking processes (Smith et al. 2014, Hoe et al. 2016). Further research is needed to test whether a global stream enhances the explanatory strength of Kingdon’s MSF in LMIC settings and how this global stream can be integrated into the theoretical framework.

## Conclusion

This study examined the NCD policy formulation process in Nepal using Kingdon’s MSF, contributing to limited empirical evidence on NCD policy formulation in LMICs. The convergence of global and local evidence, global guidance, feasible policy options, and international political commitments opened a policy window that enabled adoption of the PEN programme. The STEPS survey findings played a central role in NCD recognition, while WHO guidance and recommendations for the PEN programme provided a feasible policy option. Political engagement and global commitments further created a supportive environment for policy adoption, indicating the interaction between domestic and global forces on the policy process. The findings support the use of Kingdon’s MSF to understand the complex dynamics of NCD policy formulation in Nepal but also suggest the relevance of an additional global stream.

For researchers and policymakers who are embarking on a similar process to understand and strengthen the policy process, particularly in LMICs, the study underscores the importance of strengthening research capacity, institutionalizing multisectoral coordination, balancing global guidance with national ownership, and enhancing sustainable financial mechanisms (Table 4). These components are essential to ensure that political prioritization is translated into effective NCD policy implementation.

**Table 4:** Policy and practice recommendations.

Challenges	Possible policy response
Limited involvement of relevant stakeholders in the policy formulation process due to a lack of stakeholder analysis framework	Adopt a comprehensive stakeholder selection approach to ensure inclusiveness and engagement of relevant stakeholders.
Limited engagement of non-health stakeholders in the policy formulation process	Enhance awareness about the multi-faceted nature of NCDs, importance of addressing them, and how non-health stakeholders can contribute to addressing these issues.
Sporadic, individualized, and limited advocacy efforts for NCDs.	Promote a more systematic and coordinated approach to advocacy for NCDs.
Limited uptake of local evidence by policymakers	Establish effective mechanism to integrate local and context specific evidence in policy decisions. Improve communication and collaboration between researchers and policymakers.
Reliance on external stakeholders to formulate the policy due to limited capacity of national actors	Strengthen capacity of national stakeholders to generate, interpret, and use evidence. This approach can enable them to better contextualize policy decisions and align with both the country’s needs and global guidelines.

## Supplementary Material

czag048_Supplementary_Data

## Data Availability

The datasets generated and/or analyzed during this study are available from the corresponding author on reasonable request for academic purposes only.

## Reflexivity statement

The original data was collected by one person as part of a PhD project, and as such it needed to be their own work and was limited by time and resources. Three of the authors have over 20 years’ experience of working in Nepal, and two authors were born in Nepal, and one is Nepali. Authors come from a range of backgrounds in public health and have worked in many regions of the world. There is a gender balance in the authors, with the primary author being a female and three other authors being male. There were also far more males than females available for interview, and extra efforts were made to interview as many women as possible who were involved. However, only one female participant was interviewed. We think this reflects the paternalism of institutional structures and society in Nepal, which favours men being in positions of organizational power. Although there are no authors currently living in Nepal, the first author (A.V.) is a Nepalese PhD student conducting the research reported in this paper in Nepal whilst being registered at a UK university. P.S. is a Nepalese academic currently employed at a UK university, A.L. is a Malaysian academic currently employed at a UK university, and E.v.T. is a Dutch academic employed at a UK university.
