# Supplementary material for: Understanding the formulation of non-communicable disease policies in Nepal: a qualitative study
Source: Health Policy Plan. 2026 Apr 8;41(6):955–66. doi: 10.1093/heapol/czag048 (PMC13276260; doi:10.1093/heapol/czag048)
Supplement: czag048_Supplementary_Data [file czag048_supplementary_data.zip › Supp file 1_Strength and limitations.docx]

**Strength and limitations of Kingdon’s Multiple Streams Framework**

Over the years, Kingdon’s MSF has faced numerous criticisms. However, it has been widely applied in policy research across various regions, including the United States, Europe and Asia. Scholars from different policy fields, including health policy, have found it to be a relevant and easily applicable model (Butler, 2015; Duke et al., 2013; Dykeman & Williams, 2014; Jansson & Tillgren, 2010; Rawat & Morris, 2016). Rawat & Morris (2016) highlights the suitability of Kingdon’s framework, emphasising its remarkable flexibility, which enhances its feasibility across diverse settings while remaining valid for explaining the policy activities. Studies have demonstrated a growing preference for Kingdon’s MSF among scholars who consider it a useful lens for understanding the complex and dynamic nature of policy formation (Crowley, 2013; Jones et al., 2016; Simanjuntak et al., 2012). Furthermore, there has been an emphasis on adapting the framework to address contextual and applicability concerns. Therefore, Kingdon’s MSF has been chosen as the analytical lens for this research, which aims to understand the process of NCD policy formulation and the factors influencing this process in Nepal.

Whilst acknowledging the strength of the framework, it is also important to point out its weaknesses and the considerations made in the study. Because Kingdon’s study focused on making federal policy in the United States, it provides limited information about the dynamics between central and local governance levels (Exworthy & Powell, 2004). Hence, different studies have questioned the transferability of this framework while conducting the study at different governance levels. Conversely, some authors stress the possibility of using this framework while assessing policy processes at different government levels (Ackrill et al., 2013). This is something my research picks up as it explores the policy formulation process in the federal context of Nepal, aiming to understand how things work and how interactions take place at different levels.

The absence of institutional components in the MSF is widely criticised, and a key distinction from the Garbage Can Model (Jones et al., 2016; Zohlnhofer & Friedbert, 2016). While Kingdon focused on actors through the concept of policy entrepreneurs, the Garbage Can Model incorporated organisational context (Saetren, 2016). Spohr (2016) highlights the importance of institutions that “shape constellations of actors and their goals” as well as influencing perceptions, preferences and the feasibility of policy within the dynamics of the politics stream (Spohr, 2016, pp. 251–253). Similarly, some emphasised the need to modify Kingdon’s MSF to account for institutional factors to better understand governance autonomy, entrepreneurs’ role, and the relationship between different factors that lead to varying policy outcomes (Smith, 2018). In response to this criticism about the lack of institutional components in the MSF, I have incorporated this element as a sub-component within the framework in this study. Whilst concerns exist regarding its use, with some amendments, it can serve as a useful tool for understanding the policy making process.
