# Supplementary material for: Understanding the formulation of non-communicable disease policies in Nepal: a qualitative study
Source: Health Policy Plan. 2026 Apr 8;41(6):955–66. doi: 10.1093/heapol/czag048 (PMC13276260; doi:10.1093/heapol/czag048)
Supplement: czag048_Supplementary_Data [file czag048_supplementary_data.zip › Supplementary file 2.docx]

**Inclusion criteria of literature review**

- Population: Studies involving different stakeholders involved in the NCD policies formulation.
- Topic focus: Studies assessing formulation of four essential NCDs (Cardiovascular diseases, Chronic obstructive pulmonary disease, Diabetes mellitus, cancer) and their four main risk factors (physical inactivity, tobacco consumption, unhealthy food habits and alcohol consumption) policies in LMICs.
- Time: Studies published from 2011 to 2022.
- Language: Studies published in English.

**Exclusion criteria of literature review**

- Population: Studies involving stakeholders who were not involved in the NCD policy formulation process.
- Topic focus: Studies assessing the formulation process of the policies other than the four essential NCDs and four risk factors.
- Time: Studies published before 2011.
- Language: Studies published in other languages.
