# Supplementary material for: Understanding the formulation of non-communicable disease policies in Nepal: a qualitative study
Source: Health Policy Plan. 2026 Apr 8;41(6):955–66. doi: 10.1093/heapol/czag048 (PMC13276260; doi:10.1093/heapol/czag048)
Supplement: czag048_Supplementary_Data [file czag048_supplementary_data.zip › Supplementary file 3.docx]

**Using information power to determine samples in the study**

Information power was assessed based of five components: (i) study aim- the study had a narrow focus, exploring how NCD policy was formulated in Nepal; (ii)sample specificity- participants directly involved in the policy formulation process were purposively selected; (iii) use of established theory- application of Kingdon’s MSF enabled collection of rich information from a small sample (iv) quality of dialogue- the researcher’s prior knowledge, formal trainings in qualitative methods and guidance from more experienced supervisors, facilitated strong communication and in-depth data collection; (v) analysis strategy- a single holistic case design focusing on particularly three NCD-related policies required less sample size to offer sufficient information power compared to cross case analysis studies. Based on these components, a sample of 12 participants was deemed sufficient to provide adequate information power to address the study objectives.
