# Supplementary material for: Understanding the formulation of non-communicable disease policies in Nepal: a qualitative study
Source: Health Policy Plan. 2026 Apr 8;41(6):955–66. doi: 10.1093/heapol/czag048 (PMC13276260; doi:10.1093/heapol/czag048)
Supplement: czag048_Supplementary_Data [file czag048_supplementary_data.zip › Supplementary file 4.docx]

**Framework analysis**

In the first stage of data analysis, the audio recordings were carefully listened to, next, interview transcripts were read thoroughly to gain an in-depth understanding and to identify emerging patterns. Key issues relevant to the research questions were noted (Ritchie & Spencer 1994; Spencer et al. 2014). An a priori analytical framework based on Kingdon’s MSF was used to analyse the dataset. Relevant segments from interviews were linked to corresponding framework components, e.g. the problem stream. New framework components were created (e.g. institutional procedures) when data offered valuable insights into the policy process but did not align with the pre-defined framework. Similar codes were grouped, leading to creation of sub-components and framework components.

The coded data were linked to the framework components via appropriate units of analysis, representing the key entities under study. The interviews focused on how the problem was identified (problem stream), how the strategy to address the problem was decided (policy stream), and how the political factors influenced the policy formulation process (politics stream). Hence, these three streams were applied as the primary units of analysis for the indexing process. This indexing process was iterative involved testing and refining the framework by confirming or revising the definitions of components and sub-components, and adding new sub-components as needed (Goldsmith, 2021).

Next, the indexed data were systematically organised and summarised, and charts were created. Since the framework components already had a logical order, the data were arranged accordingly, bringing together similar patterns and categorising them as sub-components. This enhanced both the coherence and hierarchy of the framework’s structure. Finally, in the mapping and interpretation stage, insights from earlier steps were combined with observed data patterns, associations, variations, and comparisons across and within the units of analysis and framework components (Ritchie & Spencer 1994; Spencer 2014). For example, the data showed a consistent perspective among policymakers regarding the key leadership role of the Ministry of Health and Population during the policy formulation process. This analysis technique also helped to identify areas of silence and varying emphasis of the participants.
