# Supplementary material for: Understanding the formulation of non-communicable disease policies in Nepal: a qualitative study
Source: Health Policy Plan. 2026 Apr 8;41(6):955–66. doi: 10.1093/heapol/czag048 (PMC13276260; doi:10.1093/heapol/czag048)
Supplement: czag048_Supplementary_Data [file czag048_supplementary_data.zip › Supplementary file 5.docx]

**Limitations**

There were few limitations in this study. This study only included stakeholders from health sector. Non-health sector stakeholders such as those from education, agriculture, finance and so on were not involved. This was primarily due to limited access to these stakeholders as a student researcher, as well as time and resource constraints. While this inclusion allowed in-depth understanding of health sector’s role in the NCD policy formulation process, it may have overlooked the breadth of perspectives potentially on intersectoral dynamics, and facilitators and barriers to multisectoral collaboration. Since NCD prevention and control require multisectoral action, it may not have captured the broader policy context. Future research incorporating experiences of non-health sector stakeholders would provide a more holistic understanding of the policy process and help identify facilitators to strengthening multisectoral collaboration for effective NCD prevention and control in Nepal. As the policy was formulated in 2014 and 2019, the study required the participants to respond retrospectively, with some participants moved on to occupy other positions which may have led to recall bias and influenced the accuracy of their response. However, no major divergent views were witnessed in the data and was comparable to the official policy documents, maximising the data trustworthiness. Furthermore, because the participants were the elite groups affiliated to different institutions, they may have provided responses aligning with their organisational positions or expectations rather than their personal views. This could have affected the diversity of perspectives, particularly related to sensitive issues related to policy process, such as institutional performance or intersectoral collaboration. Efforts were made to reduce this bias by using the information from other participants to further explore issues, encouraging open discussions and ensuring confidentiality. Another limitation of this study is that it used a qualitative approach due to which the findings may not be generalisable to other settings due to Nepal’s unique political and socio-cultural milieu, influencing the policy environment. Hence, lessons drawn from this study may not be applicable to another context.
