# Supplementary material for: Understanding the formulation of non-communicable disease policies in Nepal: a qualitative study
Source: Health Policy Plan. 2026 Apr 8;41(6):955–66. doi: 10.1093/heapol/czag048 (PMC13276260; doi:10.1093/heapol/czag048)
Supplement: czag048_Supplementary_Data [file czag048_supplementary_data.zip › Table 1_clean.docx]

Table 1: Overview of non-communicable diseases policies

| **Policies** | **Focus** |
| --- | --- |
| National Health Policy 2014 | It focuses on universal access to health services, basic health services, health financing, governance and human resources. |
| Multisectoral action plan for prevention and control of NCDs (2014-2020) | It mainly focuses on prevention of NCDs, including lifestyle and behaviour change (tobacco and alcohol control), strengthening PHC, monitoring and supervision, and multi-sectoral coordination. |
| National Health Policy 2019 | It focuses on more comprehensive health services such as Universal Health Coverage, integrated services (promotive, curative, rehabilitative, and palliative), financing, governance, and health information systems. |
| Package of essential non communicable disease (PEN) intervention at primary health service setting: PEN training trainee’s manual | PEN interventions have four simplified protocols.   1. Protocol 1: Heart attack, stroke and kidney disease prevention via integrated management of diabetes and hypertension. 2. Protocol 2: Health education and counselling on healthy behaviours. 3. Protocol 3: Chronic Obstructive Pulmonary Disease and Asthma Management 4. Protocol 4: Assessment and referral of women with suspected cancer (breast and cervix)   The main objectives of PEN interventions are  (i) strengthen the health system to prevent and control NCDs and their risk factors through PHC services, (ii) strengthen capacity and coordination at the national and local levels for effective NCD prevention and control, (iii) Reduce NCD-related modifiable risk factors and social determinants through health promotion initiatives. |

NCDs: Non-communicable disease; PEN: Package of Essential Non-communicable diseases; PHC: Primary Health Care.
