# Supplementary material for: Understanding the formulation of non-communicable disease policies in Nepal: a qualitative study
Source: Health Policy Plan. 2026 Apr 8;41(6):955–66. doi: 10.1093/heapol/czag048 (PMC13276260; doi:10.1093/heapol/czag048)
Supplement: czag048_Supplementary_Data [file czag048_supplementary_data.zip › Table 2.docx]

Table 2: List of reviewed documents

| **Author** | **Name of the document** |
| --- | --- |
| World Health Organization, 2013a | Global action plan for the prevention and control of noncommunicable diseases 2013-2020. |
| World Health Organization, 2013b | Implementation tools: Package of essential noncommunicable (PEN) disease interventions for primary health care in low-resource settings. |
| Ministry of Health and Population, 2014 | National Health Policy 2014 |
| Government of Nepal, 2014 | Multisectoral Action Plan for Prevention and Control of Non-communicable Diseases (2014-2020) |
| Government of Nepal, 2015 | Constitution of Nepal |
| Ministry of Health and Population, 2019 | National Health Policy 2019 |
| World Health Organisation et al., 2019 | Package of Essential Non communicable Disease (PEN) intervention at primary health service setting: PEN training trainee’s manual |
| Department of Health Services, 2023 | Annual Report (2021/22) |
| Department of Health Services, 2024 | Annual Health Report 2079/80 |
| Department of Health Services, 2025 | Annual Health Report 2080/81 |
