# Supplementary material for: Understanding the formulation of non-communicable disease policies in Nepal: a qualitative study
Source: Health Policy Plan. 2026 Apr 8;41(6):955–66. doi: 10.1093/heapol/czag048 (PMC13276260; doi:10.1093/heapol/czag048)
Supplement: czag048_Supplementary_Data [file czag048_supplementary_data.zip › Table 3_clean.docx]

Table 3: Participant characteristics

| **Type of respondent** | **Total number** |
| --- | --- |
| Government actors | 8 |
| Ministry of Health and Population (1) |  |
| Government organization (2) |  |
| Research/Academic institutions (3) |  |
| Hospital (2) |  |
| Non-government actors | 4 |
| International organization (1) |  |
| NGOs/Civil Society Organization (2) |  |
| Research/Academic institutions (1) |  |

NGOs: Non-governmental organizations
