# Supplementary material for: Understanding the formulation of non-communicable disease policies in Nepal: a qualitative study
Source: Health Policy Plan. 2026 Apr 8;41(6):955–66. doi: 10.1093/heapol/czag048 (PMC13276260; doi:10.1093/heapol/czag048)
Supplement: czag048_Supplementary_Data [file czag048_supplementary_data.zip › Table 4_clean.docx]

Table 4: Policy and practice recommendations

| **Challenges** | **Possible policy response** |
| --- | --- |
| Limited involvement of relevant stakeholders in the policy formulation process due to a lack of stakeholder analysis framework | Adopt a comprehensive stakeholder selection approach to ensure inclusiveness and engagement of relevant stakeholders. |
| Limited engagement of non-health stakeholders in the policy formulation process | Enhance awareness about multi-faceted nature of NCDs, importance of addressing them, and how non-health stakeholders can contribute to addressing these issues. |
| Sporadic, individualized and limited advocacy efforts for NCDs. | Promote a more systematic and coordinated approach to advocacy for NCDs. |
| Limited uptake of local evidence by policymakers | Establish effective mechanism to integrate local and context specific evidence in policy decisions. Improve communication and collaboration between researchers and policymakers. |
| Reliance on external stakeholders to formulate the policy due to limited capacity of national actors | Strengthen capacity of national stakeholders to generate, interpret and use evidence. This approach can enable them to better contextualise policy decisions and align with both country’s needs and global guidelines. |

NCD: Non-communicable disease
