# Supplementary figures and images for: Understanding the formulation of non-communicable disease policies in Nepal: a qualitative study
Source: Health Policy Plan. 2026 Apr 8;41(6):955–66. doi: 10.1093/heapol/czag048 (PMC13276260; doi:10.1093/heapol/czag048)

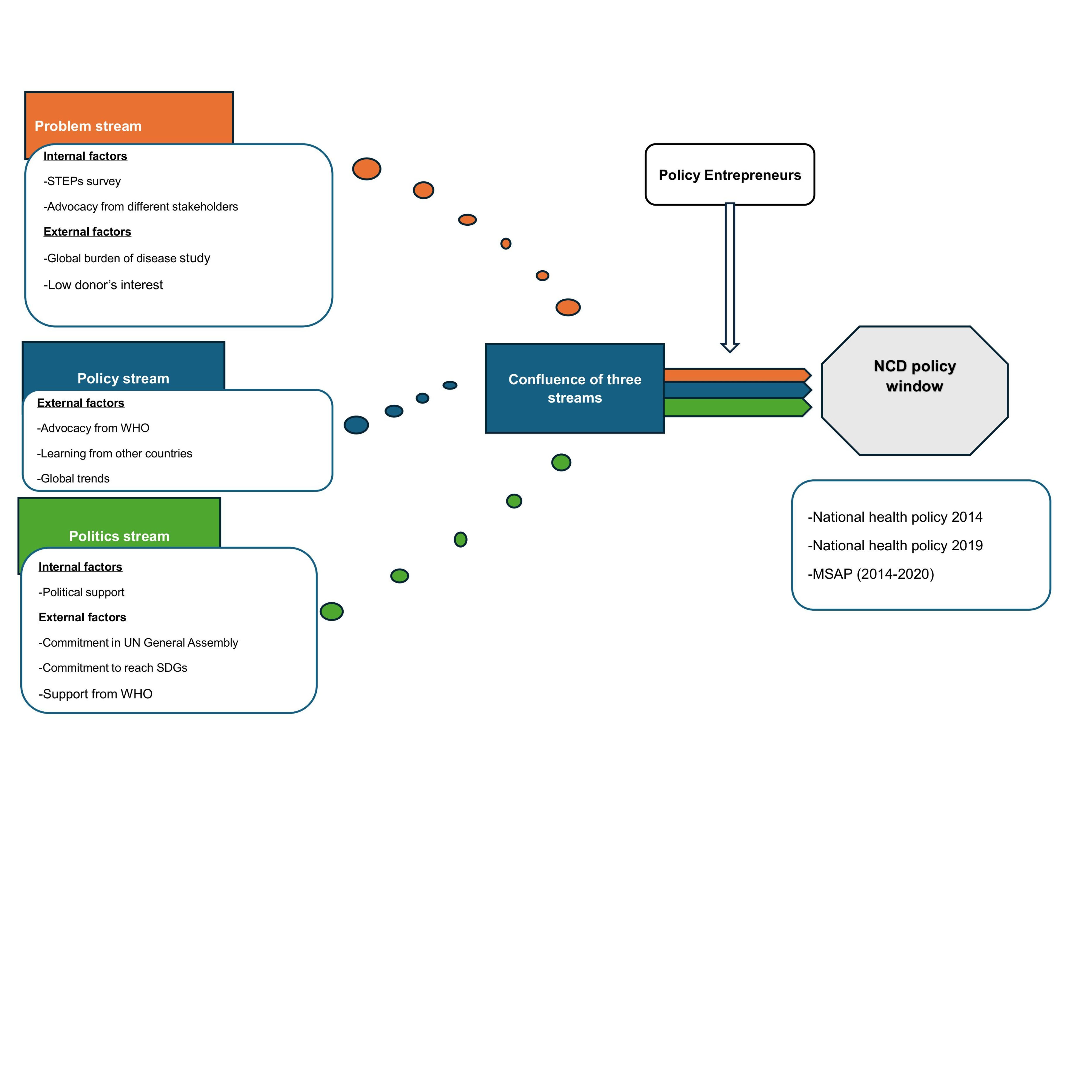

Supplement: czag048_Supplementary_Data [file czag048_supplementary_data.zip › Figure 1 300dpi.jpg]
